# Six Visual Rating Scales as A Biomarker for Monitoring Atrophied Brain Volume in Parkinson’s Disease

**DOI:** 10.14336/AD.2019.1103

**Published:** 2020-10-01

**Authors:** Yu Lin, Ying Fu, Yi-Fang Zeng, Jian-Ping Hu, Xiao-Zhen Lin, Nai-Qing Cai, Qiang Weng, Yi-Jing Zhao, Yi Lin, Dai-Rong Cao, Ning Wang

**Affiliations:** ^1^Department of Neurology and Institute of Neurology, The First Affiliated Hospital, Fujian Medical University, Fuzhou 350005, China.; ^2^Central Laboratory, The First Affiliated Hospital, Fujian Medical University, Fuzhou 350005, China.; ^3^Department of Radiology, First Affiliated Hospital, Fujian Medical University, Fuzhou 350005, China.; ^4^Department of Geriatrics, First Affiliated Hospital, Fujian Medical University, Fuzhou 350005, China.

**Keywords:** whole brain atrophy, visual rating scale, structural image, Parkinson’s disease, clinical trials

## Abstract

The focus of our investigation was to determine the feasibility of using six visual rating scales as whole-brain imaging markers for monitoring atrophied brain volume in Parkinson’s disease (PD). This was a prospective cross-sectional single-center observational study. A total of 98 PD patients were enrolled and underwent an MRI scan and a battery of neuropsychological evaluations. The brain volume was calculated using the online resource MRICloud. Brain atrophy was rated based on six visual rating scales. Correlation analysis was performed between visual rating scores and brain volume and clinical features. We found a significant negative correlation between the total scores of visual rating scores and quantitative brain volume, indicating that six visual rating scales reliably reflect whole brain atrophy in PD. Multiple linear regression-based analyses indicated severer non-motor symptoms were significantly associated with higher scores on the visual rating scales. Furthermore, we performed sample size calculations to evaluate the superiority of visual rating scales; the result show that using total scores of visual rating scales as an outcome measure, sample sizes for differentiating cognition injury require significantly fewer subjects (n = 177) compared with using total brain volume (n = 2524). Our data support the use of the total visual rating scores rather than quantitative brain volume as a biomarker for monitoring cerebral atrophy.

Brain atrophy assessment is an important biomarker in Parkinson’s disease (PD) because of its relationship to neurodegeneration and the progression of disability [1, 2]. Assessment of atrophy helps to distinguish between clinically and cognitively deteriorating patients and can predict those who will have a less-favorable clinical outcome over the long term [3]. However, there are numerous challenges to measuring brain volume in a routine clinical setting. It is well established that for reliable measurements of brain volume changes over time, patients should undergo imaging acquisition with the same scanner and without scanner/software/protocol changes. However, this is very difficult to achieve in a clinical setting. Although, several software tools are currently available and have already been applied in research or clinical trial settings for volumetric measurements of whole brain, they generally have different requirements for operator technical ability and levels of operator intervention required. Whole brain volumetric measurements are still labor-intensive and depend on specific acquisition techniques [4, 5]. Therefore, there is an urgent need for routine individual-level brain atrophy monitoring in the clinical setting.

Specifically designed visual rating scales developed to assess atrophy offer a cost-effective tool that is ideally suited for implementation in clinical practice [6]. Their sensitivity and reliability have been determined by comparing visual rating scores with volumetric data in individual subjects. Recently, Harper et al. applied six MRI visual rating scales to brain scans from 184 individuals with post-mortem-confirmed dementia and healthy controls [7]. Compared with voxel-based morphometric images, these visual rating scales were capable of providing more precise assessment for distinguishing the pattern of regional atrophy, including orbito-frontal (OF), anterior cingulate (AC), fronto-insula (FI), anterior temporal (AT), medial temporal lobe (MT), and posterior atrophy (PA). This study used a multicenter setting and MRI data with variable quality (1.5 and 3 Tesla), which suggests that the six MRI visual rating scales provide robust data compared to the aforementioned volumetric measurements [8]. Subsequent studies have also confirmed the robustness of the scale method [9, 10]. PD patients were reported to have a wide range of brain atrophy, particularly in the parietal, occipital, temporal, and frontal lobes, as well as in the hippocampus, amygdala, caudate, putamen, and thalamus. Single regional atrophy is insufficient for representing the diverse patterns of brain atrophy in PD [2, 11, 12]. Given the evidence for the patterns of brain atrophy in PD, we hypothesized that total visual rating scores should be associated with total brain volume. Therefore, we combined the six 6 visual rating scales in order to perform a comprehensive full brain atrophy analysis. We aimed to investigate the feasibility of using the six visual rating scales for brain atrophy assessment.

## MATERIALS AND METHODS:

### Subjects

This prospective cross-sectional observational study was approved by the Ethics Committee of the First Affiliated Hospital of Fujian Medical University (approval number: 2014063), and written informed consent was obtained from all participants. The patients were recruited from outpatients who met the United Kingdom Parkinson’s Disease Society Brain Bank criteria [13] and were admitted to the neurology department of First Affiliated Hospital of Fujian Medical University between 2017 and 2018. The final Parkinson’s disease diagnoses were based on both clinical and radiological findings after a review of all clinical and investigative information by a panel of neurology experts. Exclusionary criteria were atypical syndrome or secondary Parkinsonism, a history of brain surgery or trauma, unstable depression and anxiety, history of drug or alcohol abuse, unable to undergo neuropsychological assessment (eg: sever visual impairments) and MRI contraindications or poor image quality.

### Evaluations and cognitive classification

All subjects underwent detailed neurological examinations and assessments. Information was obtained from patients and their caregivers in face-to-face interviews, including demographics, clinical features, and medical and family history. The motor symptoms were assessed using Part III of the Movement Disorder Society-Unified Parkinson’s Disease Rating Scale (MDS-UPDRS) [14] and the Hoehn and Yahr stage scale (H-Y stage) on normal medication (on-stage). Cognition was evaluated by the Montreal Cognitive Assessment (MoCA) and Mini-Mental State Examination (MMSE), both of which were widely used in previous studies and were recommended by the Movement Disorder Society (MDS) Task Force for diagnose of cognitive disorders[3, 15, 16]. The non-motor symptom scale (NMSS) contains nine dimensions: cardiovascular, sleep/fatigue, mood/cognition, perceptual problems, attention/memory, gastrointestinal, urinary, and sexual function, and it is now widely used to assess the frequency and severity of NMS in PD patients across all stages in conjunction with a validated non-motor questionnaire [17]. Depression was evaluated using the Hamilton Depression Rating Scale (HAMD), which was recommended by the MDS Task Force for screening purposes and measurement of severity of depressive symptoms [18]. The Hamilton anxiety rating scale (HAMA) was used for the assessment of anxiety in PD patients. As reported by the MDS task force, HAMA fulfils the criteria for a “suggested” scale [19, 20]. The subjects who fulfilled the MDS Task Force criteria for probable PDD were classified as PD patients with dementia (PDD) or as PD patients without dementia (nPDD) [16].

### MRI Scanning

Structural MR Images were obtained on a 3.0 T MR machine (Siemens, Erlangen, Germany). The scanning sequence included an axial T1-weighted sequence, a T2 -weighted sequence, and a high-resolution 3D-T1 weighted sequence. The 3D-T1 weighted images were acquired as follows: TR = 2,300 msec; TE = 2.32 msec; TI = 900 msec; flip angle = 8°; 192 slices; field of view (FOV) = 240 × 240mm^2^; voxel size = 0.9 × 0.9 × 0.9mm^3^.

**Figure 1.**
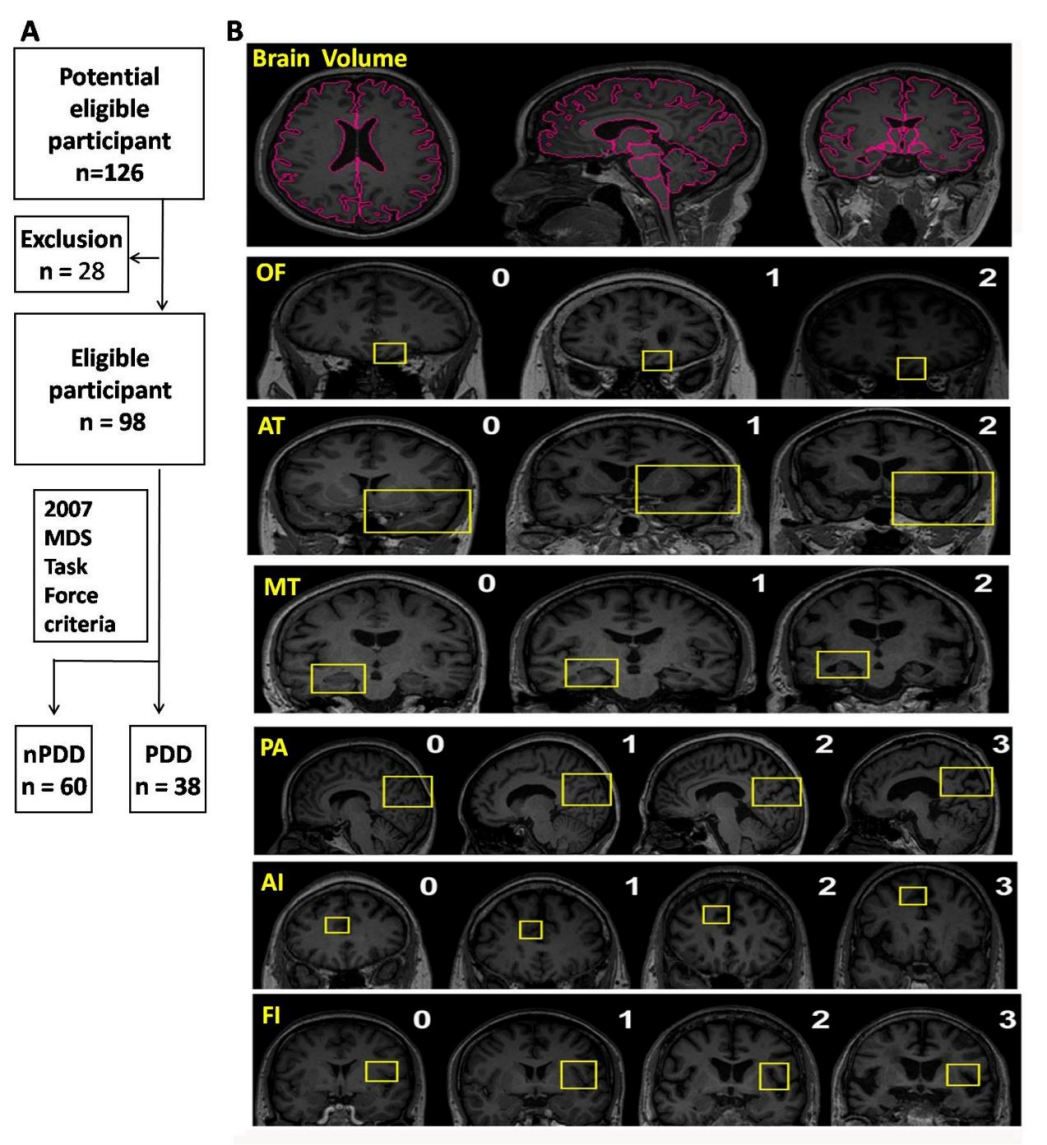
Trial profile. (A) Diagrammatic sketch of the screening process; (B) Brain volume as quantified by MRICloud and examples of scoring of six visual rating scales. Red coloring indicates the segmentation region at level 1. The yellow frame indicates each region for image assessment. PDD and PD patients with dementia; nPDD and PD patients without dementia. OF = orbitofrontal cortex; AC = anterior cingulate; FI = frontoinsula; AT = anterior temporal; MT= medial temporal lobe; PA = posterior cortex.

### Quantitative analysis of brain volume

The 3DT1-weighted images were converted from DICOM format to Analyzed format using a DICOM-to-Analyze converter offered by MRICloud. The analysis of 3DT1-weighted images was then performed using the open online resource MRICloud (www.mricloud.org) [21, 22]. The whole brain was segmented into different structures and were grouped into ﬁve levels of granularity based on their ontological relationships without second segmentation, and the detailed protocol was used from a previous study [23]. The large atlas database combined with the dynamic age-matching approach has been shown to produce improved segmentation accuracy and reproducibility. The atlas version Adult50_90yrs_2 83Labels_26atlases_M2_V9B was selected to best match the individuals in the study. Statistics of the volume from each brain parcel were obtained from downloaded segmentation results. All brain volumes were linearly normalized to the MNI space. The total brain volume was calculated by summing up the volume of grey matter (GM) and white matter (WM) based on the aforementioned segmentation results at level 1 of granularity (Fig. 1).

Visual rating of cerebral atrophy

Visual rating of the 3D-T1 sequence of all individuals was performed by two specially trained neurologists who were blind to all clinical information. The axial, sagittal, and coronal planes were inspected using the ROIEditor software, which is available at www.mristudio.org. Six regions were rated that span from the anterior to the posterior, including the orbitofrontal cortex (OF), anterior cingulate (AC), fronto-insula (FI), anterior temporal (AT), medial temporal lobe (MT), and posterior cortex (PA) (Fig. 1). Detailed rating protocol were used from an earlier publication. Briefly, six visual rating scales were modified from Harper et al. and reference images were provided. For each scale, slice selection was specified to improve consistency. For the OF and AC, the olfactory sulcus and cingulate sulcus were rated on the same slice where the corpus callosum becomes visible. For the FI, the circular insular sulci were rated on the slice where the anterior commissure become visible, as well as the two posterior slices. For the MT, structures including the hippocampus, temporal horn, and choroid ﬁssure were observed over several slices. For the AT, the temporal sulci and temporal lobe were rated on the coronal slice where the connection between the frontal and temporal lobes become invisible. The PA assessment was focused on structural changes involving the precuneus, posterior cingulate sulcus, the parietal lobe, and the parieto-occipital sulcus when scrolled through in the posterior direction [7].

### Statistical analysis

Statistical analyses were performed using the Statistical Package for Social Sciences (SPSS, Inc., Chicago, IL, USA, version 22). A two-tailed P value of <0.05 was considered significant. The Kolmogorov-Smirnov test was been used to assess the normality of the data. The intraclass correlation coefficient (ICC) was used to evaluate inter-rater reliability of two raters and the reproducibility of brain volume analysis based on the MRICloud platform. A two-way random model with absolute agreement was selected for this study.

Step 1: Correlation analysis between visual rating scores and brain volume: since the data were nonnormally distributed, the spearman rank correlation test was performed between the total scores of six visual rating scales and brain volume for all subjects. Furthermore, a Pearson partial correlation test was performed for controlling for age, sex, and educational level.

Step 2: Relationship between visual rating scales and clinical features: the correlation between total visual rating scores and clinical features (NMSS, MoCA, MDS-UPDRS-III, and HAMD) were determined using multiple linear regression models. Age, sex, and education level were used as covariates.

Step 3: Sample sizes for differentiating cognition injury: the sample size required for differentiating cognition injury per arm was calculated for a hypothetical clinical trial. The total scores of visual rating scales and the brain volume were compared. The calculation was based on the assumption that cognitive state of PD patients changed from non-cognition injury to cognition injury; an 80% power level was used, and a one-side 0.05 level was considered significant. The formula used to calculate sample size per trial arm is as follows: n = 2σ^2^(z_1-α_+z_1-β_)^2^ /μ_1_-μ_2_)^2^, where z_1-β_= 0.84 to provide 80% power, z_1-β_= 1.65 to test at the 5% significance level, and μ_1_ and μ_2_ are the mean of the total scores of visual rating scales or the brain volume, respectively. σ^2^ is the common variance of the total scores of visual rating scales or the brain volume in both arms.

## RESULTS

### Demographic and clinical data

A total of 126 patients with PD were screened from 2016 through 2018, and 98 patients were enrolled in the study; reasons for exclusion are detailed in Figure 1. There were 46 women and 52 men with a mean age of 60.1 years. The disease evolution mean was 4.4 years, and the modified Hoehn and Yahr stage median score was 2.5; this included 38 patients with dementia (PDD) and 60 patients without dementia (Table1).

**Table 1:** Demographic and clinical features of PD patients.

Patient number	98
Mean age (SD), year	60.1 ± 8.7
Sex, male, n (%)	52 (53)
Median education (range), year	8 (0-15)
Mean duration (SD), year	4.4 ± 4.6
Median MDS-UPDRS-III (range)	35.5 (10-107)
Median H-Y stage (range)	2.5 (1-4)
Median LEDD (range), mg/day	300.0 (0-1048.8)
Median HAMD (range)	5 (0-43)
Median HAMA (range)	4 (0-30)
Median NMSS (range)	27 (0-140)
Median MMSE (range)	27 (13-30)
Median MoCA (range)	23 (5-30)
Mean brain volume (SD), 10^3^mm^3^	1,180.5 (98.8)
Median total visual rating scores (range)	11 (2-20)

MDS-UPDRS = Movement Disorder Society-Unified Parkinson’s Disease Rating Scale; H-Y stage = Hoehn and Yahr stage scale; LEDD = levodopa equivalent daily dose; MoCA = Montreal Cognitive Assessment; MMSE = Mini-Mental State Examination; HAMD = Hamilton Depression Rating Scale; HAMA = Hamilton Anxiety rating scale; NMSS = non-motor symptom scale.

### Inter-rater reliability of visual rating scores

Inter-rater agreement (determined for 79 MR examinations by 2 raters) for each rating scale was excellent, with the value of ICC varying between 0.71 and 0.94. The results are presented in Supplementary Table 1.

### Inter-rater reliability of brain volume analysis

In order to further ascertain the reproducibility of brain volume analysis based on MRICloud platform, ten 3DT1 images were segmented a second time. The value of ICC was 0.99 based on two segmentations.

### Feasibility of six visual rating scales for brain atrophy measurements

**Figure 2.**
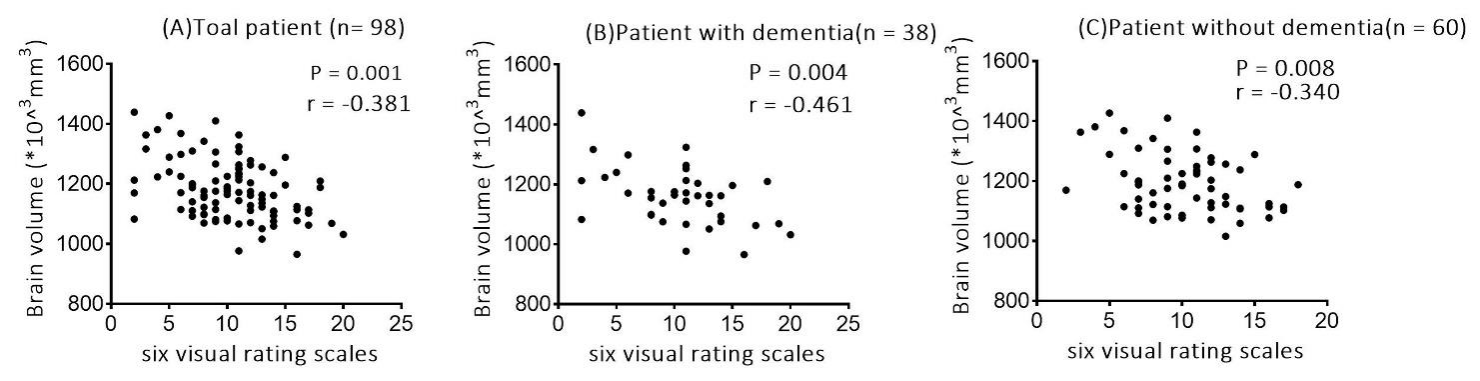
Scatter-plots showing the associations between total visual rating scores and brain volume in the total PD sample and subset group. (A) Total PD samples, (B) Patients with dementia, (C) Patients without dementia. Data were analyzed using the spearman rank correlation test.

To determine if the combination of six visual rating scales sufficiently reflect whole-brain atrophy, we performed correlation analysis between the total scores of six visual rating scales and the quantitative brain volume. A significant negative correlation was found between the total visual rating scores and the brain volume (r = -0.381 P = 0.001) (Fig. 2A). Based on evidence that brain volume varies with age, sex, and education level [24], a further partial correlation was performed; the correlation remained significant (r = -0.295, P = 0.004) after controlling for age, sex, and education. In addition, there were still significant negative correlations between the total visual rating scores and the brain volume in subgroups with cognitive impairment (r = -0.461, P = 0.004) and without (r = -0.340, P_2_= 0.008) (Fig. 2B-C). The results indicate that higher scores of six visual rating scales correlate with smaller brain volumes, regardless of cognitive impairment.

**Table 2:** Association between total score of six visual rating scales and functional, cognitive, and psychological injury.

	Unadjusted value		Adjusted value	
	Beta (95% CI)	P-value	Beta (95% CI)	P-value
UPDRS-III	0.6 (-0.1-1.3)	0.105	0.4 (-0.4-1.3)	0.302
NMSS	2.2 (0.7-3.8)	0.005	2.4 (0.7-4.2)	0.008
MoCA	-0.1 (-0.4-0.2)	0.399	-0.1 (-0.2-0.3)	0.638
HAMD	0.3 (-0.1-0.7)	0.160	0.5 (0.1-1.0)	0.026

CI = confidence interval; UPDRS-III = the part III of Movement Disorder Society-Unified Parkinson’s Disease Rating Scale; MoCA = Montreal Cognitive Assessment; MMSE = Mini-Mental State Examination; HAMD = Hamilton Depression Rating Scale; The multiple linear regression models were used for outcome and adjusted for age, sex, and/or education, n = 98 in this analysis.

**Figure 3.**
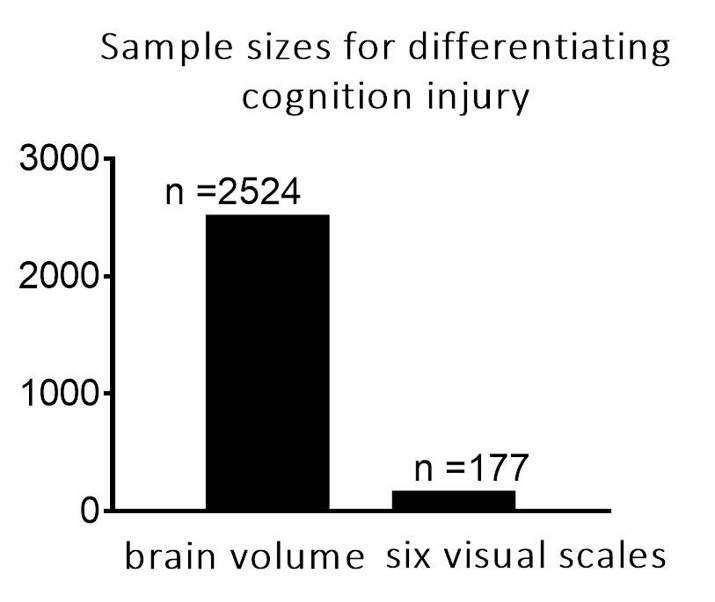
Sample size per treatment arm using brain volume and visual rating scales. The calculation was based on the assumption that cognitive state of PD patients changed from non-cognition injury to cognition injury; an 80% power level was used, and a one-side 0.05 level was considered significant.

### Association between visual rating scales with clinical features

To examine the relationship between scores of visual rating scales and clinical features, we performed multiple linear regression-based analyses, and the results are presented in Table 2. Multiple linear regression-based analysis exhibited statistically significant positive correlations between the total scores of six visual rating scales and the scores of the NMSS (β = 2.2, P = 0.005). After adjusting for age, sex, and education, the associations remained significant (β = 2.4, P = 0.008). No significant correlation was found between total visual rating scores and MDS-UPDRS-III (β = 0.6, P = 0.105) or MoCA (β = 0.3, P = 0.16). When adjusted for age and sex, the HAMD analysis revealed a positive correlation with total visual rating scores (β = 0.5, P = 0.026). The results suggest that more severe non-motor symptoms were significantly associated with higher scores on the visual rating scales.

### Sample size and differentiating cognition injury

To evaluate the superiority of visual rating scales compared with quantitative methods for future hypothetical clinical trials, we performed sample size calculations for differentiating cognition impairment based on the total scores of visual rating scales and brain volume. The results shown in Figure 3 demonstrate that using scores of visual rating scales as an outcome measure requires significantly fewer subjects (n = 177) compared with brain volume as outcome measures (n = 2524).

## DISCUSSION

The importance of assessing whole brain atrophy using rating scores in clinical trials, together with the rather limited scope of previous studies, motivated our investigation. We included 98 PD patients who underwent an MRI scan and a battery of neuropsychological evaluations. We found a significant negative correlation between the total scores of six MRI visual rating scales and quantitative brain volume using the MRICould method. The negative correlation remained significant after controlling for age, sex, and level of education. Moreover, similar negative correlations were found in subgroups with and without dementia. Both a visual rating scale and brain volume analysis was performed in rare studies[7, 9, 10]. In previous studies, the visual rating of atrophy was confined to the region considered for evaluating the application value of visual scales in the diagnosis of dementia diseases[9, 10]. Our study however tried to evaluate the validation of visual scales for the exploration of whole brain atrophy in clinical trials in PD patients.

A valid biomarker as a surrogate outcome in a clinical trial must be correlated with the clinical outcome and be sensitive enough to track disease progression [25]. This study also elucidated the clinical relevance of the combination of six scales. Our findings of multiple linear regression-based analyses exhibited statistically significant positive correlations between the total scores of six visual rating scales and scores of the NMSS and HAMD. These results indicate brain atrophy detected by six visual rating was significantly associated with non-motor symptoms, especially depression, and these results are in broad agreement with previous studies [26-30]. Thobois et al. reported that PD-associated depression is linked with temporal atrophy, particularly in the amygdala and hippocampus [26]. The atrophy of orbitofrontal cortex in PD patients has also been correlated with the severity of depression [27-30]. It is widely accepted that depression is one of the most common non-motor symptoms associated with PD and may even occur in the premotor stage of the disease [31]. The current study provides cross-sectional evidence that the six visual rating scales can function as MRI biomarkers for neuropsychological dysfunction even in early stages of PD.

Despite evidence for the association between the six visual rating scales and non-motor symptoms, multiple linear regression-based analyses exhibited no statistically significant correlations between the total scores of six visual rating scales and scores of the MoCA and UPDRS III. The relatively small sample size of this study may explain these results since we estimated sample sizes for clinical trials using the combination of rating scales versus whole brain volume outcomes. Our preliminary data indicated that multiple visual scores could provide adequate power to clinical trials with far smaller samples of patients than are required if volume measurements are used. This observation can be explained by the fact that sample sizes increase with the square of the SD of the rate of change of measurements in the relevant clinical group [25]; the smaller variance in the visual scores group is the key reason for the smaller sample sizes. The current sample size estimates are based on the simplifying assumption that a given treatment could result in a decreased cognitive state for PD patients from non-cognitive injury to cognitive injury. Such calculations are important because they show that the dimensions of clinical trials using whole brain volume as surrogate outcomes needs to significantly change once investigators aim to demonstrate beneficial effects on cognitive functioning in PD. Based on our preliminary sample size calculation, further research with larger sample sizes (more than 177 samples) may provide the optimal cut off values for detecting PD patients with and without cognitive impairment in clinical practice. A limitation of our study is the possible overestimation of the results due to the relatively small sample and cross-sectional study design. It is unclear whether this method can be performed to monitor the progression of brain atrophy, and this approach needs to be tested in a longitudinal study. Besides, PD is a general brain disorder, which is caused by the dysfunction of the entire basal ganglia-cortex-cerebellum system [32]. As a result, we suspect that the six visual rating scales are inadequate as a biomarker for general brain atrophy of PD without cognition injury due to the mild sensibility of reflecting atrophy in relatively small subcortical structures.

In conclusion, our study identified six visual rating scales that reliably reflect whole brain atrophy in patients with PD. Since visual rating scales are both quick and easy to apply, and can be performed on routinely acquired images, this method can be used as a potential means for monitoring cerebral atrophy in clinical practice.

## Supplementary Materials

The Supplemenantry data can be found online at: www.aginganddisease.org/EN/10.14336/AD.2019.1103.
