# Supplementary material for: Six Visual Rating Scales as A Biomarker for Monitoring Atrophied Brain Volume in Parkinson’s Disease
Source: Aging Dis. 2020 Oct 1;11(5):1082–90. doi: 10.14336/AD.2019.1103 (PMC7505277; doi:10.14336/AD.2019.1103)
Supplement: Supplementary file 1 — The Supplemenantry data can be found online at: www.aginganddisease.org/EN/10.14336/AD.2019.1103. [file AD-11-5-1082-suppl.pdf]

SUPPLEMENTARY DATA

Six Visual Rating Scales as A Biomarker for Monitoring Atrophied Brain Volume in Parkinson's Disease

Yu Lin[1,#], Ying Fu[1,2,#], Yi-Fang Zeng[1,#],Jian-Ping Hu[3], Xiao-Zhen Lin[4], Nai-Qing Cai[1], Qiang Weng[3], Yi-Jing Zhao[3], Yi Lin[1], Dai-Rong Cao[3*], Ning Wang[1*]

[1]Department of Neurology and Institute of Neurology, The First Affiliated Hospital, Fujian Medical University, Fuzhou 350005, China.
[2]Central Laboratory, The First Affiliated Hospital, Fujian Medical University, Fuzhou 350005, China.
[3]Department of Radiology, First Affiliated Hospital, Fujian Medical University, Fuzhou 350005, China.
[4]Department of Geriatrics, First Affiliated Hospital, Fujian Medical University, Fuzhou 350005, China.

© 2019. Lin Y et al. Published online at http://www.aginganddisease.org/EN/10.14336/AD.2019.1103

Supplementary Table 1. Assessment of consistency of rating between raters.

**Visual rating scales**	**Intraclass correlation coefficient (ICC)**
Total visual rating scales	0.94
OF_L	0.81
OF_R	0.71
AC_L	0.76
AC_R	0.87
AT_L	0.81
AT_R	0.72
FI_L	0.82
FI_R	0.72
MT_L	0.76
MT_R	0.71
PA_L	0.82
PA_R	0.77

L = Left; R = Right; OF = orbitofrontal cortex; AC = anterior cingulate; FI = frontoinsula; AT = anterior temporal; MT= medial temporal lobe; PA = posterior cortex.

© 2019. Lin Y et al. Published online at http://www.aginganddisease.org/EN/10.14336/AD.2019.1103
